# Children's Use of Electronic Games: Choices of Game Mode and Challenge Levels

**DOI:** 10.1155/2010/218586

**Published:** 2010-06-27

**Authors:** Cindy H. P. Sit, Jessica W. K. Lam, Thomas L. McKenzie

**Affiliations:** ^1^Institute of Human Performance, The University of Hong Kong, Pokfulam, Hong Kong; ^2^School of Exercise and Nutritional Sciences, San Diego State University, 5500 Campanile Drive, CA 92182-7251, USA

## Abstract

*Introduction*. Interactive electronic games are popular and are believed to contribute to physical activity accrual. The purpose of this study was to examine children's electronic game use during conditions in which they had free access to selecting interactive and seated screen-based versions of electronic games and during the interactive versions had free choice in making adjustments to the activity intensity. *Methods*. We systematically observed 60 Hong Kong primary school children during two 60-minute game sessions while simultaneously recording their game mode choices and physical activity levels using SOFIT (System for Observing Fitness Instruction Time). *Results*. When given free choice, children spent more than half of their available time participating in interactive versions of games. These versions of games provided significantly more moderate-to-vigorous physical activity and greater energy expenditure than the computer screen versions. Children with the opportunity to modify intensity levels spent more time playing the interactive versions and accrued more physical activity. *Conclusions*. The tenets of behavioral choice theory were supported. Access to new-generation interactive games, particularly those with modifiable intensity levels, may facilitate children's participation in physical activity.

## 1. Introduction

Current health guidelines recommended that children should participate in 60 minutes of moderate-to-vigorous physical activity (MVPA) daily [1]. Children, however, often adopt sedentary lifestyles and there is widespread concern that screen-based media such as computer and video games contribute to sedentary living and childhood obesity problems [2, 3]. New generation interactive electronic games, known as exergaming, have been developed and are considered as a promising way to promote physical activity in children. Previous studies have demonstrated that interactive electronic games can significantly increase physical activity in children [4–6], including eliciting greater energy expenditure compared to seated electronic games [7–12]. Boys are typically found to spend more energy during interactive games than girls [6, 7], but sometimes no significant gender effects are reported [8, 10]. Compared to overweight children, nonoverweight children have been shown to be more willing to play an interactive dance game [13]. These studies, however, have typically assessed children's physical activity levels during short-time periods (e.g., 15 minutes per game segment), and without participants having a choice of the interactive or more sedentary computer screen-based versions of the same game. 

Direct observation exceeds other measures of physical activity in providing contextually-rich data on the environment [14, 15]. Several studies have used this method with cohorts of children in their homes in Hong Kong [16] and the USA [17, 18] and have shown that children spend most of their leisure time indoors and in sedentary pursuits. Using the behavioral choice theory [19–21] as a conceptual framework, Sit et al. [22] recently used direct observation to study electronic game behavior during extended time periods (i.e., 60 minutes continuously) and under conditions when children had choices in playing interactive or computer screen versions of the same games—bowling and running. Findings showed that children spent about half their time on the interactive versions of games and that these versions engaged children in substantially MVPA more than during computer screen versions (i.e., 70% versus 2% of game time). Additionally, boys and nonoverweight children expended more energy during the interactive games than girls and overweight children, respectively. The study concluded that children, when given the opportunity (i.e., availability and accessibility), tended to select interactive games over the more sedentary versions of the same sport game. 

Reducing sedentary behavior, in particular that associated with screen-time media, continues to be an important goal in childhood obesity prevention and treatment. The present investigation extended the previous study [22] to examine children's electronic game behavior under more complex conditions. Thus, in addition to being able to select sedentary or physically active versions of the same sport games, children were given immediate access to adjusting the levels of challenge (i.e., activity intensity) during the interactive game modes. We hypothesized that giving access to adjusting levels of challenge within interactive games would increase both children's time playing the games and their physical activity levels over the seated versions of the same sport game. We also predicted that boys would spend more time and expend more energy during interactive games than girls.

## 2. Methods

### 2.1. Participants

Sixty healthy primary children (35 boys, 25 girls) aged between 9 and 12 (*M* = 10.77 ± 0.79) were recruited from local primary schools. Informed written consent from children and assent from parents were obtained prior to commencement of the study. Ethical approval was granted by the University of Hong Kong.

### 2.2. Design and Procedures

The children participated alone in two 60-minute sessions in a controlled laboratory setting. They were allowed to choose and play either an interactive electronic game or a similarly themed computer screen game. They were also allowed to switch between game modes and to stop playing at any time. During session one, the children could select between bowling-type games (i.e., interactive bowling called XAviX Bowling) and. during session two, select between running-type games (i.e., interactive running game called Aerostep), both developed by Shiseido Co., Japan (http://www.xavix.com/). Additionally, the children were randomized by computer into two groups, stratified by gender. One group (i.e., “fixed intensity”) played the interactive games in a preset mode while children in the second group (i.e., “adjustable intensity”) were permitted to freely modify game difficulty (i.e., intensity) levels. Prior to the study none of the children had previously played either the interactive or computer screen versions of games. All sessions were video-taped for reliability checks and data analyses. 

The children were familiarized with the laboratory, the procedures, and the interactive and computer-based versions of the games prior to the game sessions. Anthropometric data were measured using standard practice by a certified technician. Weight was measured to the nearest 0.1 kg and height to the nearest 0.1 cm using a freestanding Seca stadiometer (Seca AG, Reinach, Switzerland). Bioimpedance (TBF-401, Tanita Co., Japan), after controlling for hydration and skin temperature in the air-conditioned laboratory, was used to estimate fat mass (kg), fat free mass (kg), and percent body fat.

### 2.3. Observation System

A modification of the System for Observing Fitness Instruction Time (SOFIT) instrument [23] was used to determine each child's physical activity and the amount of time he/she spent in each game mode during each of the two 60-min sessions. Physical activity was recorded using momentary time sampling by entering one of five codes every 20 seconds: lying down (code 1), sitting (code 2), standing (code 3), walking (code 4), or vigorous (code 5). These codes have been validated using heart rate monitoring and accelerometry [24–26]. Assessors were trained to use SOFIT following the standard protocol [14], which included memorizing coding definitions and conventions, viewing video segments, and surpassing the interobserver agreement (IOA) of 85% on video assessments prior to data collection. Reliability assessments were performed for 20% of the total data, and IOA for child physical activity levels exceeded 99%.

### 2.4. Data Analysis

Dependent variables were mean minutes children spent in each game mode, time spent in different physical activity levels, and estimated energy scores. Child physical activity variables were expressed as both minutes per session and as the proportion of session time. The Walking and Vigorous categories were summed to form Moderate to Vigorous Physical Activity (MVPA), a description often used in health-related literature, and when converted to percentage of time serves as a measure of physical activity intensity. In addition, a summary score for estimated energy expenditure during sessions and game modes, Total Energy Expenditure (TEE) (kcal/kg), was obtained using standard calculations based on heart rate monitoring [14]. Independent variables were gender and the interactive game level groupings (fixed, adjustable).

Data were analyzed using SPSS 16.0, and descriptive statistics, including means, standard deviations, frequencies, and percentages, were obtained for all variables. One-way ANOVAs were conducted to test for significant group and gender differences for game modes (i.e., mean minutes for each game mode) and physical activity variables (i.e., the five codes, plus MVPA%, and TEE). Partial Eta Squared (*n*_*p*_^2^) statistics were used to indicate the relative magnitude of the differences between group means: small = 0.01, medium = 0.06, and large = 0.14 [27]. Chi-square analysis was performed to identify the frequency distribution of group and gender by body weight classification based on Cole et al.'s [28] work. Data checks were performed prior to data analyses to ensure no violation of the assumptions of normality, linearity, homogeneity of variances, homogeneity of regression slopes, and reliable measurement of the covariate. Alpha level was set at *P* < .05 for all statistical tests.

## 3. Results

### 3.1. Physical Characteristics of Participants by Group and Gender

Table 1 shows that the adjustable intensity level group and boys had significantly greater body weight, BMI, and BMI *z* score than the fixed intensity level group and girls, respectively. Based on the International Obesity Task Force (IOTF) definitions of child obesity [28], 22 participants were overweight (18 boys, 4 girls) and 9 were obese (8 boys, 1 girl). Results of chi-square statistics (data not shown) indicated that a significantly greater proportion of adjustable intensity level group was overweight or obese than control group, *N* = 60, *χ* = 5.41, *P* ≤ .05. More boys were found to be overweight or obese than girls, *N* = 60, *χ* = 17.21, *P* ≤ .0001. Because BMI was a confounding variable, one-way between-groups ANCOVAs, adjusting for BMI, were performed for subsequent analyses. 

### 3.2. Time Spent during Game Sessions

**Figure 1:**
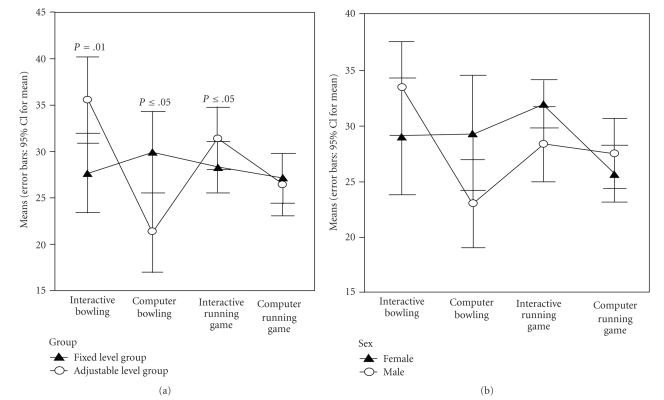
Mean minutes spent by game type and mode, after adjusting for BMI.

Children spent nearly all of their allocated 60-minute sessions playing the available games (bowling games, 95.5% of the time; running games, 94.5% of the time). Overall, the children spent the largest amount of time playing the interactive bowling game, followed by the interactive running game, computer screen running game, and computer screen bowling game. Figure 1 shows the mean minutes spent in each game mode by group and gender, after adjusting for BMI. Compared to the fixed intensity level interactive game group, the adjustable intensity level group spent significantly more time in interactive bowling (i.e., 7.8 minutes), *F*(1, 57) = 6.70, *P* = .01, *n*_*p*_^2^ = .011; and interactive running games (i.e., 3 minutes), *F*(1, 57) = 5.34, *P* ≤ .05, *n*_*p*_^2^ = .09; but less time in computer screen bowling, *F*(1, 57) = 6.31, *P* ≤ .05, *n*_*p*_^2^ = .11. No significant gender differences in the four game modes were noted. 

### 3.3. Levels of Physical Activity during Game Sessions

Table 2 presents the mean minutes and proportion of time children spent in the five activity codes during each game mode. In general, when playing computer screen games, children spent over 95% of their time sitting. In contrast, they spent 77% of their time (25.1 minutes) walking during interactive bowling and 83.8% (25.1 minutes) of their time in vigorous activities during interactive running games. 

Results (data not shown here) indicated that the adjustable intensity level group was more physically active than the fixed level group, spending more time walking, *F*(1, 57) = 10.47, *P* ≤ .05, *n*_*p*_^2^ = .16; and less time standing, *F*(1, 57) = 15.32, *P* ≤ .0001, *n*_*p*_^2^ = .21; during interactive bowling. The adjustable level group also spent less time sitting during computer screen bowling, *F*(1, 57) = 7.23, *P* ≤ .05, *n*_*p*_^2^ = .11; and computer screen running games, *F*(1, 57) = 4.55, *P* ≤ .05, *n*_*p*_^2^ = .07. Compared to girls, boys spent more time in vigorous activities during interactive bowling, *F*(1, 57) = 6.78, *P* ≤ .05, *n*_*p*_^2^ = .11; and interactive running games, *F*(1, 57) = 3.69, *P* ≤ .05, *n*_*p*_^2^ = .06. Girls, conversely, spent more time standing, *F*(1, 57) = 6.74, *P* ≤ .05, *n*_*p*_^2^ = .11; and walking, *F*(1, 57) = 3.94, *P* ≤ .05, *n*_*p*_^2^ = .07; during interactive running games. 

Table 3 presents summary scores for children's physical activity levels during game modes in terms of MVPA percent and TEE. Overall, MVPA percent and TEE were substantially higher during interactive games than their computer screen versions, with the interactive running game producing the highest values. 

Compared to the fixed intensity level group, the adjustable level group had greater MVPA percent during interactive bowling, *F*(1, 57) = 9.33, *P* ≤ .05, *n*_*p*_^2^ = .14; computer screen bowling, *F*(1, 57) = 6.32, *P* ≤ .05, *n*_*p*_^2^ = .10; and computer screen running games, *F*(1, 57) = 5.10, *P* ≤ .05, *n*_*p*_^2^ = .08. Similarly the adjustable intensity level group also had higher TEE during interactive bowling, *F*(1, 57) = 9.78, *P* ≤ .05, *n*_*p*_^2^ = .15; and during interactive running games, *F*(1, 57) = 5.31, *P* ≤ .05, *n*_*p*_^2^ = .09; but lower TEE during computer screen bowling, *F*(1, 57) = 5.90, *P* ≤ .05, *n*_*p*_^2^ = .09. No significant gender differences in activity variables were evidenced. 

## 4. Discussion

A main aim of the present study was to determine whether access to adjusting difficulty levels (intensity) influences the amount of time and the activity levels in children playing interactive versions of the games. Consistent with an earlier study by Sit et al. [22], when given free choice, children spent about 95% of each allocated hour session playing games. During sessions, they chose to spend over half their time playing interactive games over more sedentary, computer screen versions. The children were substantially more physically active during interactive (88% MVPA) than computer screen (4% MVPA) versions of games, and this is congruent with previous studies which illustrated exergaming can contribute substantially to children's physical activity levels [4–12].

Children in the adjustable intensity level group, who had free access to modifying levels of challenge/intensity in interactive games, spent more time playing the interactive versions of games (bowling, 7.8 more min; running games, 3 more min) than those in the fixed intensity level group. Additionally, they had a higher MVPA percent during interactive bowling and greater TEE during both interactive bowling and running games. These results support the notion that children are attracted by a feature of exergaming that challenges them to engage in physically active behavior while simultaneously decreasing their sedentary computer screen-time behavior [6]. The results also support the tenets of behavioral choice theory, given that the availability of exergaming offered an appealing alternative to sedentary computer screen-based media and that immediate access to different physically active alternatives (i.e., game level adjustments) acted as reinforcers to sustain interactive game play. Previous intervention studies have reported that interactive electronic games are able to motivate children to be more physically active over time [29, 30].

Contrary to previous studies [6, 7], we found no significant gender differences in the overall amount of time spent in each of the game modes or in MVPA percent and TEE. Boys, however, engaged in a greater proportion of time in vigorous activities during interactive games. In particular, boys spent 85.8% (versus 81% for girls) of their time in engaging vigorous activities during interactive running. This suggests that the physically demanding nature of interactive running game may be more attractive to boys than girls. A confounding event, however, is that the interactive running game required children to mimic “Jackie Chan” (a famous Hong Kong male marital artist) and to engage in a 5-minutes workout continuously. Male combatants would appear periodically and children could make them disappear by quickly stepping on places on a mat. The male characters in the game content might be more attractive to boys, which might, in turn, reinforce and motivate them to exert more effort [31]. Nonetheless, and inconsistent with our previous study [22], game mode selection and activity patterns were similar for boys and girls, suggesting that the availability and accessibility of game level adjustments have a generalizable effect on influencing children's electronic game behavior.

Overall, the current study provides additional evidence to suggest that making interactive games available may reduce the amount of time children spend in sedentary pursuits [19–21]. Built-in challenges based on increased complexity and intensity levels have the potential for engaging children in interactive games for longer periods of time and at higher activity levels. A definite strength of most interactive games is their ability to be customized in a way to challenge individuals. Because children spend large amounts of time at home and are typically sedentary when observed there [16–18], interactive games have potential to provide opportunities for increasing physical activity in that location. 

Limitations of the study include a small sample size, assessing children's physical activity in a controlled setting. and examining only two paired interactive and computer screen electronic games. Future research should focus on larger sample sizes, using additional objective measures of physical activity, and assessing the longer-term outcomes of interactive games interventions on children's physical activity.

## Figures and Tables

**Table 1:** Physical characteristics of participants (Mean ± SD).

Variable	All	Interactive game group		Gender	
	*N* = 60	Fixed *n* = 30	Adjustable *n* = 30	Boys *n* = 35	Girls *n* = 25
Age	10.8 ± 0.8	10.9 ± 0.9	10.6 ± 0.6	10.7 ± 0.7	10.8 ± 0.9
Height (cm)	146.3 ± 8.1	145.8 ± 8.3	146.7 ± 8.0	146.0 ± 8.7	146.6 ± 7.4
Weight (kg)	44.4 ± 12.4	41.0 ± 11.7^a^	47.7 ± 12.3^a^	48.2 ± 13.1^a^	39.0 ± 9.1^a^
BMI (kg/m²)	20.5 ± 4.5	19.0 ± 4.1^b^	22.0 ± 4.4^b^	22.3 ± 4.4^c^	18.0 ± 3.3^c^
*z*BMI (kg/m²)	0.0 ± 1.0	−0.2 ± 1.0^b^	0.5 ± 1.0^b^	0.6 ± 1.0^c^	−0.4 ± 0.8^c^

BMI:Body Mass Index.

Significant mean differences between independent variable categories:

^a^ *P* ≤ .05, ^b^ *P* ≤ .001, ^c^ *P* ≤ .0001.

**Table 2:** Mean proportion of session time ± SD (and mean min ±SD) for activity levels during game type and mode.

Activity	Interactive bowling	Computer bowling	Interactive running game	Computer running game
	mean min = 31.6 ± 12.5	mean min = 25.7 ± 12.3	mean min = 29.9 ± 8.3	mean min = 26.8 ± 8.0
Lying Down %	0.0 ± 0	0.0 ± 0	0.0 ± 0	0.0 ± 0
	(0.0 ± 0)	(0.0 ± 0)	(0.0 ± 0)	(0.0 ± 0)
Sitting %	5.4 ± 6.4	95.7 ± 4.1	0.9 ± 2.3	95.3 ± 6.0
	(2.0 ± 2.5)	(24.9±12.4)^a^	(0.3 ± 0.6)	(25.7±8.2)^a^
Standing %	16.6 ± 23.9	0.6 ± 1.5	0.7 ± 1.4	0.4 ± 0.8
	(4.2±7.0)^b^	(0.1 ± 0.2)	(0.2±0.4)^c^	(0.1 ± 0.2)
Walking %	77.0 ± 22.6	3.7 ± 3.3	14.6 ± 7.8	3.4 ± 2.8
	(25.1±13.3)^a^	(0.7±0.6)^a^	(4.3±2.7)^c^	(0.8 ± 0.6)
Vigorous %	1.0 ± 2.7	0.0 ± 0	83.8 ± 8.5	0.9 ± 4.3
	(0.3±0.8)^c^	(0.0 ± 0)	(25.1±7.6)^c^	(0.2 ± 0.9)

Significant group difference after adjusting for BMI: ^a^ *P* ≤ .05, ^b^ *P* ≤ .0001,

Significant gender difference after adjusting for BMI: ^c^ *P* ≤ .05.

**Table 3:** Children's overall mean MVPA% ± SD (actual min ± SD) and TEE by game type and mode.

Physical activity levels	Interactive bowling	Computer bowling	Interactive running game	Computer running game
MVPA % (min)				
All	78.0 ± 22.8	3.7 ± 3.3	98.4 ± 2.6	4.3 ± 5.6
	(25.4 ± 13.4)	(0.7 ± 0.6)	(29.4 ± 8.3)	(1.0 ± 1.1)
Group				
Fixed level	(69.0±28.0)^a^	(2.7±2.5)^a^	98.2 ± 2.4	(2.6±2.6)^a^
	(19.3 ± 12.2)	(0.6 ± 0.4)	(26.9 ± 7.2)	(0.6 ± 0.5)
Adjustable level	(87.0±11.4)^a^	(4.7±3.7)^a^	98.4 ± 2.7	(6.0±7.2)^a^
	(31.4 ± 12.1)	(0.8 ± 0.7)	(31.9 ± 9.2)	(1.3 ± 1.4)
Gender				
Boys	82.1 ± 20.9	3.9 ± 3.5	98.5 ± 3.0	4.3 ± 6.1
	(28.3 ± 13.1)	(0.7 ± 0.6)	(28.6 ± 9.7)	(1.0 ± 1.0)
Girls	72.3 ± 24.9	3.3 ± 3.1	98.2 ± 1.9	4.3 ± 4.9
	(21.4 ± 13.1)	(0.7 ± 0.5)	(30.6 ± 5.3)	(1.0 ± 1.2)

TEE				
All	2.76 ± 1.2	1.24 ± 0.6	4.05 ± 1.1	1.32 ± 0.4
Group				
Fixed level	2.28 ± 1.1^a^	1.43 ± 0.5^a^	3.71 ± 1.0^a^	1.31 ± 0.3
Adjustable level	3.24 ± 1.2^a^	1.06 ± 0.5^a^	4.39 ± 1.3^a^	1.32 ± 0.4
Gender				
Boys	3.01 ± 1.2	1.13 ± 0.5	3.97 ± 1.4	1.35 ± 0.4
Girls	2.42 ± 1.2	1.39 ± 0.6	4.17 ± 0.7	1.27 ± 0.3

MVPA:Moderate to Vigorous Physical Activity (walking + vigorous).

TEE:Total Energy Expenditure (kcal·kg^−1^).

Significant group mean difference after adjusting for BMI: ^a^ *P* ≤ .05.
